# Integrated Bottom-Up and Top-Down Proteomics of Patient-Derived Breast Tumor Xenografts* [Fn FN2]

**DOI:** 10.1074/mcp.M114.047480

**Published:** 2015-10-26

**Authors:** Ioanna Ntai, Richard D. LeDuc, Ryan T. Fellers, Petra Erdmann-Gilmore, Sherri R. Davies, Jeanne Rumsey, Bryan P. Early, Paul M. Thomas, Shunqiang Li, Philip D. Compton, Matthew J. C. Ellis, Kelly V. Ruggles, David Fenyö, Emily S. Boja, Henry Rodriguez, R. Reid Townsend, Neil L. Kelleher

**Affiliations:** From the ‡Proteomics Center of Excellence,; §Department of Chemistry, and; ¶Department of Molecular BiosciencesNorthwestern University, Evanston, IL 60208;; ‖Department of Internal Medicine, Washington University School of Medicine, St Louis, MO 63110;; **Department of Molecular & Cellular Biology, Baylor College of Medicine, Houston, TX 77030;; ‡‡Center for Health Informatics and Bioinformatics, and Department of Biochemistry and Molecular Pharmacology, New York University Medical School, New York, NY 10016;; §§Office of Cancer Clinical Proteomics Research, National Cancer Institute, Bethesda, MD 20892

## Abstract

Bottom-up proteomics relies on the use of proteases and is the method of choice for identifying thousands of protein groups in complex samples. Top-down proteomics has been shown to be robust for direct analysis of small proteins and offers a solution to the “peptide-to-protein” inference problem inherent with bottom-up approaches. Here, we describe the first large-scale integration of genomic, bottom-up and top-down proteomic data for the comparative analysis of patient-derived mouse xenograft models of basal and luminal B human breast cancer, WHIM2 and WHIM16, respectively. Using these well-characterized xenograft models established by the National Cancer Institute's Clinical Proteomic Tumor Analysis Consortium, we compared and contrasted the performance of bottom-up and top-down proteomics to detect cancer-specific aberrations at the peptide and proteoform levels and to measure differential expression of proteins and proteoforms. Bottom-up proteomic analysis of the tumor xenografts detected almost 10 times as many coding nucleotide polymorphisms and peptides resulting from novel splice junctions than top-down. For proteins in the range of 0–30 kDa, where quantitation was performed using both approaches, bottom-up proteomics quantified 3,519 protein groups from 49,185 peptides, while top-down proteomics quantified 982 proteoforms mapping to 358 proteins. Examples of both concordant and discordant quantitation were found in a ∼60:40 ratio, providing a unique opportunity for top-down to fill in missing information. The two techniques showed complementary performance, with bottom-up yielding eight times more identifications of 0–30 kDa proteins in xenograft proteomes, but failing to detect differences in certain posttranslational modifications (PTMs), such as phosphorylation pattern changes of alpha-endosulfine. This work illustrates the potency of a combined bottom-up and top-down proteomics approach to deepen our knowledge of cancer biology, especially when genomic data are available.

Recent advances in high-throughput genomics have allowed deep characterization of cancer at the DNA and RNA level. Large-scale initiatives, such as The Cancer Genome Atlas at the National Cancer Institute, have provided comprehensive genomic analyses of human tumors from many cancer types and, thus, the prospect for novel insights into the pathways leading to cancer and new possibilities for medical advances. It is well known that genomic aberrations and an inability to properly maintain and repair genetic material enable tumor initiation and progression (1). The large-scale mapping of cancer genomes has provided a detailed catalogue of mutations and polymorphisms that may translate into proteome variation and has left researchers wondering which genomic abnormalities drive tumor biology and which are functionally irrelevant. Although RNA sequencing can provide supporting evidence for the translation of DNA-level mutations into the proteome and alternative splicing, events, including signal peptide cleavage and a multitude of biologically active posttranslational modifications (PTMs) can significantly increase protein variation that RNA-seq data could not reliably predict. Recent studies have also shown that RNA transcript measurements poorly predict protein abundance differences between tumors (2). Thus, detection of mutations and PTMs at the protein level provides a direct readout of the biological impact of cancer-related genomic abnormalities.

Proteomic technologies, especially those based on mass spectrometry (MS), have the potential to detect genetic aberrations at the protein level. These technologies aim to identify the genes that give rise to proteins, characterize any modifications from the primary amino acid sequence, and quantify differences in relative expression levels between samples. Ideally, these techniques would be operable for all the proteins expressed in a cell, tissue, or other complex protein mixture; however, this is not the case. Different technologies exist, each with its unique strengths and weaknesses. Two forms of proteomics analyses are shotgun bottom-up (BU)^1^ and top-down (TD) (3). In BU proteomics, the proteins are digested with a protease, such as trypsin, prior to peptide detection and sequencing using tandem mass spectrometry. Protease digestion results in a complex mixture of peptides between 500–3,500 Da that are usually separated by reverse phase liquid chromatography or multidimensional chromatography in-line with a mass spectrometer (4, 5). Precursor mass measurements, along with MS/MS fragmentation information, allow inference of the protein composition of the sample via these peptides. Extremely sensitive BU methods have been developed and are capable of identifying >5,000 protein groups within a single sample, with some peptide sequences present in multiple proteins or isoforms. Such shared peptides can lead to ambiguities in identifying the unique proteins present in the sample, the so called protein parsimony problem (6). Also, enzymatic digestion can result in the loss of information about combinatorial PTMs and sequence variants.

Top-down (TD) proteomics, on the other hand, does not rely on the use of proteases and examines proteins as a whole. In doing so, top-down proteomics can fully characterize the composition of individual proteoforms (7), including proteolysis products, signal peptide cleavage, sequence variants, and PTMs co-occurring on the same molecule. A typical TD workflow consists of single or multi-step protein separations, such as reverse-phase liquid chromatography (8) and GELFrEE (9), and the resulting protein fractions are further separated by liquid chromatography in line with a mass spectrometer. Advances in MS instruments and protein separations have allowed TD proteomics to become a robust technique for the identification and characterization of ∼2,000–3,000 proteoforms (8–10). Unlike BU, TD proteomics routinely links proteins to their parental genes without the problem of protein inference.

With the recent advent of methods for differential quantitation using TD on proteins below 30 kDa (11), it is now possible to begin comparing BU and TD techniques for three primary proteomic tasks: gene identification, whole proteoform characterization, and detection of differential expression. While some efforts have explored the complementarity of BU and TD technologies in the study of less complex proteomes (12, 13) and the structural analysis of antibodies (14, 15), herein we describe the first evaluation of the complementarity of BU and TD technologies for the qualitative and quantitative analysis of cancer proteomes. To accomplish this task, we employed two samples from patient-derived xenografts (PDXs) established from a basal-like (WHIM2-P32) and luminal B (WHIM16-P33) breast cancer (16–18). Patient-derived breast cancer xenografts have been established as reliable models of human tumors that provide a renewable resource for studying the human disease (16, 19, 20). These patient-derived xenograft tumor lines are genomically well-characterized (16, 17) and have been used to generate Comparison Reference (CompRef) samples within the Clinical Proteomic Tumor Analysis Consortium (CPTAC) (21) for performance validation of mass spectrometry protocols and workflows. Genome and RNA sequencing of the xenografts has provided us with lists of sequence variants, due to single nucleotide polymorphisms (SNPs), and novel splice junctions. Using these well-characterized xenograft models, we compared and contrasted the performance of BU and TD proteomic approaches to detect cancer-specific aberrations at the peptide and proteoform levels and to measure differential expression of proteins and proteoforms.

This work represents the first large-scale integration of genomic, BU, and TD proteomic data for comparative analysis of PDXs comprised of the studies described in Table I. In brief, Study 1 was designed to provide information on the ability to detect tumor-specific features informed by prior RNA-seq data of these samples (16, 17). Study 2 tested the applicability of the recently established label-free top-down quantitative proteomics platform (11) for the analysis of tumors. Finally, Study 3 sought to detect differential expression of proteins and proteoforms between basal and luminal B breast cancer samples for the low molecular weight proteome (<30 kDa).

## EXPERIMENTAL PROCEDURES

### 

#### 

##### Sample Preparation

Cryopulverization of tumor xenografts was performed at Washington University in St. Louis using the established protocols of CPTAC as previously described (22). One of the driving motivations for creating the CompRef samples was to evaluate the capacity for mass spectrometry protocols to consistently provide both qualitative and quantitative data between samples. The two Washington University Human-in-Mouse (WHIM) models chosen for this purpose represent two subtypes of breast cancer with very different intrinsic biologies (17, 18). WHIM2 is derived from a basal-like (ER-, PR+, Her2-) breast cancer whereas WHIM16 is derived from a luminal B (ER+, PR+, Her2-) breast cancer (16, 17). To prepare the samples, tumors were harvested from established xenografts, pooled, and subjected to cryopulverization to create two different homogeneous samples, P32 (WHIM2) and P33 (WHIM16). The pulverized tissue from each CompRef sample (263 mg WHIM16, P33) and (257 mg WHIM2, P32) was solubilized in 1,200 μl or 1,100 μl lysis buffer (4% sodium dodecyl sulfate, 100 mm Tris-HCl, pH 7.5) supplemented with 50 mm DTT, 10 mm sodium butyrate, and phosphatase and protease inhibitors (Thermo, Rockford, IL). The samples were then sonicated using a Covaris S220X focused ultrasonicator (Covaris, Woburn, MA) set to peak incident power (PIP) = 100, duty factor (DF) = 10, cycles/burst (CPB) = 500, duration = 60 s at 6 °C. The protein concentrations determined using the Advanced Protein Assay (Cytoskeleton, Denver, CO) were 12.7 mg/ml and 11.2 mg/ml for P32 and P33, respectively. Samples were frozen at −80 °C and shipped to Northwestern University on dry ice.

GELFrEE separation was performed as previously described (23, 24). Briefly, 400 μg of protein were precipitated with cold acetone to remove salts and suspended in 4% SDS solution prior to the addition of GELFrEE loading buffer. Separation was achieved using a commercial GELFREE 8100 fractionation system (Expedeon, Cambridge, UK) with either 8 or 10% cartridges to isolate proteins in ∼5 kDa bins from 3.5 kDa to ∼100 kDa (Supplemental Fig. S1). SDS was removed using the method described by Wessel and Flügge (25), unless otherwise noted. Tumor samples were centrally prepared at Northwestern University, some of which were shipped back to Washington University in St. Louis, MO for BU proteomic analyses.

##### Bottom-Up Proteomics

##### Endoprotease Digestion of GELFrEE Fractions

The proteins in GELFrEE fractions for Study 1 were precipitated using acetone. Protein pellets were dissolved in 20 μl of Tris buffer (100 mm, pH 8.5) containing 8 m urea. GELFrEE fractions for Study 3 were received as protein pellets and dissolved in 20 μl of Tris buffer (100 mm, pH 8.5) containing 8 m urea. Horseradish peroxidase (1 μg) was added to each digest for Study 1 and Study 3 samples as a digest standard. The proteins were reduced using TCEP (5 mm) (Thermo) for 30 min, and alkylated with iodoacetamide (40 mm) (Sigma) at room temperature in the dark for 30 min. The reaction was quenched with DTT (20 mm) (Sigma) for 15 min. The methods described by Zybailov *et al.* (26) were followed with minor modifications. Specifically, the proteins were digested for 4 h with endoprotease LysC (5 μg) (Sigma) on a Thermomixer (750 rpm) at 37 °C. The digests were then diluted fourfold with Tris buffer (100 mm, pH 8.5) and trypsin (5 μg) was added with continued incubation overnight. Due to the different protein concentrations of the individual GELFrEE fractions, the enzyme to protein ratio for the LysC and trypsin digests ranged from 1:25–1:50 and 1:5–1:10, respectively. The digests were acidified to 5% formic acid (Fluka) and filtered through a Microcon centrifugal filter (30K molecular weight cutoff) (Millipore). The peptides were desalted in parallel on Glygen Nutips containing C4 and graphitic carbon solid phase on a Biomek NXP (Beckman Coulter), as previously described (27). The eluted peptides were dried in a SpeedVac and dissolved in water/acetonitrile/formic acid (98%/1%/1%) and transferred to autosampler vials for storage at −80 °C prior to LC-MS analysis.

##### High-Performance Liquid Chromatography with High-Resolution Tandem Mass Spectrometry

A NanoLC 2D Plus System with a cHiPLC-Nanoflex and AS2 autosampler (ABSciex, Concord, ON) was configured with two columns in parallel. One cHiPLC column (ChromXP C_18_ (200 μm × 15 cm; particle size 3 μm, 120 Å) was used to inject calibrant solution (β-galactosidase peptides (625 pmol/vial)), and the other cHiPLC column was used for sample analysis. The calibrant solution (500 fmol) was injected in solvent A (water/acetonitrile/formic acid, 98%/1%/1%). The samples were loaded in a volume of 10 μl at a flow rate of 0.8 μl/min followed by gradient elution of peptides at a flow rate of 800 nl/min. The calibrant solution was eluted with the following gradient conditions with solvent B (water/formic acid/acetonitrile, 1%/1%/98%):0, 2%; 3 min, 2%; 73 min, 50%; 83 min, 80%; 86 min, 80%; 87 min 2%; and 102 min, 2%. The digests from the five fractions from 0–30 kDa (Study 3) were analyzed under the following gradient conditions (time, percentage solvent B): 0, 2%; 5 min, 2%; 365 min, 35%; 400 min, 80%; 405 min, 2%; and 425 min, 2%. The digests from the 12 GELFrEE fractions (Study 1) were analyzed under the following gradient conditions (time, percentage solvent B): 0, 2%; 5 min, 2%; 650 min, 35%; 695 min, 80%; 700 min, 2%; and 720 min, 2%.

Data acquisition was performed with a TripleTOF 5600+ mass spectrometer (AB SCIEX, Concord, ON) fitted with a PicoView Nanospray source (PV400) (New Objectives, Woburn, MA) and a 10 μm Silica PicoTip emitter (New Objectives) for bottom-up proteomics. Data were acquired using an ion spray voltage of 2.9 kV, curtain gas of 20 psi, nebulizer gas of 25 psi, and an interface heater temperature of 175 °C. The MS was operated with a resolution of greater than or equal to 25,000 (fwhm) for TOF-MS scans. For data-dependent acquisition, survey scans were acquired in 250 ms from which 100 product ion scans were selected for MS2 acquisition for a dwell time of 20 ms. Precursor charge state selection was set at +2 to +5. The survey scan threshold was set to 100 counts per second. The total cycle time was fixed at 2.25 s. Four time bins were summed for each scan at a pulser frequency value of 15.4 kHz through monitoring of the 40 GHz multichannel time to digital converter detector with four-anode/channel detection. A rolling collision energy was applied to all precursor ions for collision-induced dissociation as described in the Analyst software.

The raw LC-MS data (*.wiff) were converted to *.mzML format utilizing the AB SCIEX MS Data Converter v1.3 (AB SCIEX, Concord, ON) within PEAKS STUDIO 7.0 (Bioinformatics Solutions Inc., Waterloo, Canada) (28). The resulting files were used for database searching by the PEAKS software using the following databases. Tumor-specific protein sequence databases were created by starting with RefSeq release 50 and adding variants detected in whole genome sequencing of the xenografts and the corresponding germline. Alternative splice forms detected by RNA-seq of the tumors were also added to the protein sequence database. The variant calling for the whole genome sequencing data was done using GATK version 2.6, and the RNA-seq data were analyzed using TopHat version 2.0.3. The searches were conducted with trypsin cleavage specificity, allowing three missed cleavages, oxidation of methionine and carbamidomethylation of cysteine as variable and fixed modifications, respectively. A parent ion tolerance of 25 ppm and a fragment ion tolerance of 100 millimass units were used. The MS2-based peptide identifications were validated within PEAKS software using a modified target decoy approach, decoy fusion, to estimate the false discovery rate (FDR). A 1% FDR for peptide spectral matches was used as the quality filter to identify peptides and an FDR of <0.1% for proteins with at least two unique peptides. A spectral count of 3 within the same LC/MS run was used as a quality threshold for peptides identified as resulting from SNPs or alternative splicing events. The bottom-up data were quantified using spectrum counting. The spectral counting was done using in-house developed scripts for label-free quantitation.

##### Top-Down Proteomics

##### LC/MS

For all studies, proteins were resuspended by pipetting vigorously with 40 μl solvent A (95% water, 5% acetonitrile, 0.2% formic acid) after SDS removal. Resuspended protein fractions (5 μl) were injected onto a trap column (150 μm inner diameter × 3 cm) using an autosampler (Thermo Dionex). For Study 1, a nanobore analytical column (75 μm inner diameter × 15 cm) was coupled to the trap in a vented tee setup. Upstream of the column a 15 μm spray tip from New Objective was connected. The trap and analytical column were packed with polymeric reverse phase (PLRP-S, Phenomenex) media (5 μm d_p_, 1,000 Å pore size). The Dionex Ultimate 3000 system was operated at a flow rate of 2.5 μl/min for loading samples onto the trap. Proteins were separated on the analytical column and eluted into the mass spectrometer using a flow rate of 300 nl/min and the following gradient: 5% B at 0 min.; 15% B at 5 min.; 55% B at 55 min.; 95% B from 58–61 min.; and 5% B from 64 to 80 min. Solvent A consisted of 95% water, 5% acetonitrile and 0.2% formic acid, and solvent B consisted of 5% water, 95% acetonitrile, and 0.2% formic acid. In Studies 2 and 3, proteins were injected onto a PepSwift trap column (200 μm inner diameter × 5 mm, Thermo Fisher) at 10 μl/min, separated onto a monolithic ProSwift RP-4H analytical column (100 μm inner diameter × 50 cm) and eluted into the mass spectrometer using a flow rate of 1 μl/min and the following gradient: 1% B at 0 min.; 55% B at 55 min.; 95% B from 58–61 min.; and 5% B from 64 to 80 min.

MS data were obtained on an Orbitrap Elite (Thermo) mass spectrometer fitted with a custom nanospray ionization source. Previous studies (10, 29) have demonstrated that high-energy collisional dissociation results in higher number of identifications than other fragmentation techniques, such as electron transfer dissociation and thus, high-energy collisional dissociation was the fragmentation of choice in the work described here. For proteins of molecular weight <30 kDa, the MS method included the following events: (1) FT scan, four microscans, *m/z* 500–2,000, 120,000 resolving power at *m/z* 400 and (2) data-dependent MS/MS on the top two peaks in each spectrum from scan event 1 using higher-energy collisional dissociation with normalized collision energy of 25, isolation width 50 *m/z*, four microscans, and detection of ions with resolving power of 60,000 (at *m/z* 400). For proteins of molecular weight >30 kDa, the MS method included the following events: (1) precursor scan, ion trap, 25 microscans, *m/z* 500–2,000 and (2) data-dependent MS/MS on the top two peaks in each spectrum from scan event 1 using high-energy collisional dissociation with normalized collision energy of 25, isolation width 200 *m/z*, four microscans, and detection of ions with resolving power of 60,000 (at *m/z* 400).

##### Study 1: Qualitative BU-TD Comparison of CompRef Samples (Multiple Fractions up to 100kDa)

A 10% GELFrEE cartridge was used to obtain 12 protein fractions ranging in molecular weight from 0 to 100 kDa for each tumor sample. After SDS removal, proteins were resuspended in solvent A and injected onto the PLRP-S LC setup described above. Each fraction was analyzed in triplicate, resulting in a total of 72 RAW files.

##### Data Analysis

ProSightPC PUF files were created using a custom version of the cRAWler application. These neutral mass data were searched against an eight-step search tree (Supplemental Fig. S2). First, each target was searched with strict search criteria (mass tolerance of 2.2 Da for precursor mass and 10 ppm for fragment masses) against a mouse-specific database (UniProt Release 2014_05) to remove proteins that were a good match to the murine xenograft host. This implies that proteins with identical sequence in both human and mouse will be filtered from further analysis. The filtered proteins are listed in Supplemental Table 1 under the heading Search 0. Next, a WHIM-specific PTM-annotated database was created according to the workflow in Supplemental Fig. S3. Any target failing to be identified with the mouse search was then searched against this database, first with a strict absolute mass search (*i.e.* mass tolerances of 2.2 Da for precursor mass and 10 ppm for fragment masses), followed by a strict biomarker search (*i.e.* mass tolerance of 10 ppm for precursor and fragment masses). This step identified proteoforms from the xenograft that were a good match to non-sample-specific proteoforms. The remaining unidentified proteoforms were then searched against sample-specific databases to identify proteoforms that are uniquely associated with genetic events in each of the WHIM2 and WHIM16 tumor samples. Lastly, still unidentified targets were subjected to broad searches designed to identify previously unknown proteoforms. Supplemental Table 1 lists the number of targets identified at each of these steps.

##### Study 2: Label-Free Top-Down Quantitation (Single Fraction up to 30 kDa)

An 8% GELFrEE cartridge was used to obtain a single fraction containing proteins of MW from 0 to 30 kDa. After SDS removal, proteins were resuspended in solvent A and injected onto the RP-4H LC setup described above. The GELFrEE was performed three times for each CompRef sample and the resulting protein fractions were analyzed in six LC/MS replicates for a total of 18 RAW files per sample. Neutral mass data were created and searched as described in Study 1 above, and quantitative results generated by the same analysis of variance (ANOVA) analysis described in Study 3 below.

##### Study 3: Quantitative BU-TD Comparison of CompRef Samples (Multiple Fractions up to 30 kDa)

A 10% GELFrEE cartridge was used to obtain five protein fractions ranging in molecular weight from 0 to 30 kDa for each sample. After SDS removal, proteins were resuspended in solvent A and injected onto the RP-4H LC setup described above. The GELFrEE was performed three times for each CompRef sample and the resulting protein fractions were analyzed in five LC/MS replicates, for a total of 150 RAW files. The research design is illustrated in Supplemental Fig. S4.

##### Data Analysis

The RAW files (150) generated for this study were analyzed in two steps: quantitation and proteoform identification. To identify proteoforms, for each MS1-based mass group, neutral masses were determined from all 150 RAW files, and ProSightPC PUF files were created using a custom version of the cRAWler application. These neutral mass data were searched as described above for Study 1. In the quantitation step, neutral masses were inferred from all files, and then only those with identifications from tandem MS were grouped based on accurate MS1 mass and retention time. Next, the intensity from the mass groups, for each proteoform, were standardized within each fraction. Specifically, the average intensity for all measurements of a given proteoform was subtracted from each measurement, and the resulting difference was divided by the standard deviation of all measurements of that proteoform. Subtracting the mean centers the proteoform intensity data on zero, and the division rescales the data into units of standard deviations. The standardized values were then subjected to a hierarchical linear model-based ANOVA, with Benjamini and Hochberg FDR correction at α = 0.05, to find proteoforms that were differentially expressed between WHIM2 and WHIM16.

##### TD and BU Comparisons

TD measures intact proteoforms, while BU measures tryptic peptides derived from sets of proteoforms sharing amino acid sequences. In order to consistently compare these two techniques, we chose to call sets of peptides from BU that map to a single RefSeq identification (ID) number as detecting a “protein.” Likewise, TD frequently identified more than one proteoform associated with a single RefSeq ID. Therefore, the number of proteoforms reported from each study is greater than the number of proteins identified by TD; if five proteoforms associated with a single RefSeq identifier were discovered with TD, this was reported as one protein and five proteoforms. In this study, unlike TD, BU analysis of CompRef sets allowed comprehensive identification of protein groups without any MW restriction. All of the primary mass spectrometry data are deposited at the CPTAC Data Coordinating Center as raw files for public access (https://cptac-data-portal.georgetown.edu).

## RESULTS

**Fig. 1.**
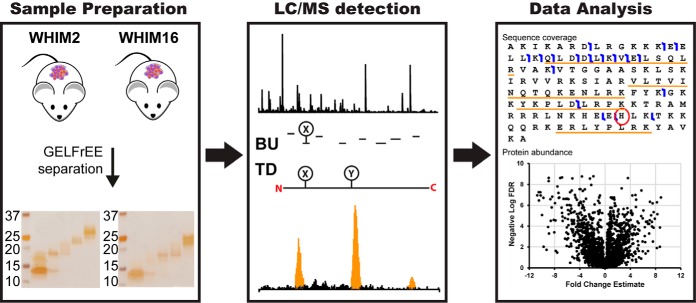
**Workflow for the qualitative and quantitative analysis of CompRef tumor xenografts by bottom-up and top-down.**

We set out to compare the ability of TD and BU to (1) identify the genes from which protein products were derived; (2) characterize proteoforms, including any PTMs, SNPs, and novel splice junctions; and (3) detect differential expression of proteins and proteoforms between a basal-like (WHIM2) and a luminal B (WHIM16) breast tumor xenograft sample. The three studies employed are described in Table I. The workflow for all three studies included GELFrEE separation prior to LC/MS and data analysis as illustrated in Fig. 1. It was expected that TD would identify fewer molecular entities but that these would be characterized proteoforms, while BU would identify a greater number of proteins but do so with lower sequence coverage.

**Table I:** Summary of experiments comparing the performance of TD and BU proteomics to detect and quantify cancer specific aberrations

Study	Description	Bottom-up	Top-down
1	Qualitative comparison of WHIM2 and WHIM16 (BU/TD) protein MW range 0–100 kDa^a^	10,453 proteins^b^ (82,156 peptides) 197 SNPs/11 NSJs^d^	2,006 proteoforms (370 proteins^c^) 5 SNPs/0 NSJs
2	Label-free TD quantitation of WHIM2 vs WHIM16 protein MW range 0–30 kDa^a^	N/P^e^	1,334 proteoforms (218 proteins^c^) 3 SNPs/1 NSJs
3	Quantitative comparison of WHIM2 and WHIM16 protein MW range 0–30 kDa^a^	3,367 proteins^b^ (49,185 peptides) 41 SNPs / 11 NSJs^d^	3,125 proteoforms (438 proteins^c^) 7 SNPs/1 NSJs

^a^ Proteins were fractionated using GELFrEE. Representative fractionations for each study are illustrated in Supplemental Fig. S1.

^b^ The term proteins corresponds to protein groups as defined by Peak Studio, ver. 7.

^c^ the term proteins corresponds to a single RefSeq identifier.

^d^ Identification required a spectrum count of 3 within a single LC/MS run.

^e^ not performed.

### 

#### 

##### Protein Identifications

Studies 1 and 3 offered head-to-head comparisons of TD and BU. Using GELFrEE to separate the proteome into molecular weight bins, we obtained information about proteins present with molecular weights ranging from 0–100 kDa and 0–30 kDa in Studies 1 and 3, respectively. The number of protein and proteoform identifications is enumerated in Table I and a detailed list is included in Supplemental Tables 1 and 2, for TD and BU, respectively. In all TD analyses, any proteoform that was consistent with mouse, even when having sequence homology with human, as in the case of histones, was removed from the counts. In both studies, BU resulted in a greater number of protein identifications than TD, as anticipated. Despite significant improvements for top-down proteomics in discovery mode (9), BU outperforms TD in the >40 kDa range.

##### Identification of Sequence Variants and Alternative Sequences Unique to the WHIM Tumor Samples

**Fig. 2.**
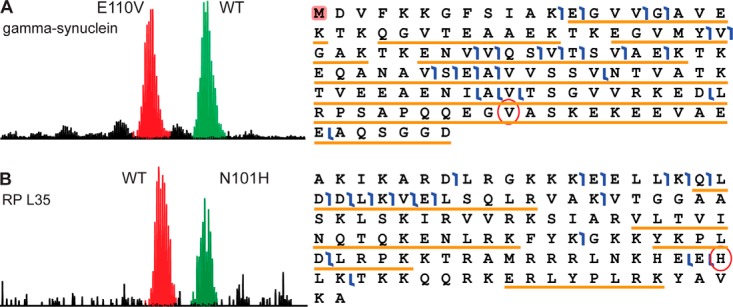
**Protein identifications in WHIM2 and WHIM16.** (*A*) TD spectrum of gamma-synuclein displaying the distinctive pattern of a heterozygote genotype at this locus, and sequence of gamma-synuclein including fragment ions (flags) detected by TD and peptide sequences (underlined) detected by BU. The highlighted N-terminal amino acid indicates an N-terminal acetylation. The cSNP E110V is circled. Both technologies provided evidence of the cSNP. (*B*) TD spectrum of ribosomal protein L35 displaying the distinctive pattern of a heterozygote genotype at this locus, and sequence of ribosomal protein L35 including fragment ions (flags) detected by TD and peptide sequences (underlined) detected by BU. The cSNP N101H is circled. Only TD provided evidence of the cSNP.

Whole genome and RNA sequencing of the WHIM2 and WHIM16 PDX models, as well as the corresponding primary tumor sample (16, 17) provide an excellent foundation for evaluating the proteomic technology capacity for detecting sample or “WHIM-specific” coding SNPs and alternate splice variants that may give rise to unique proteoforms. The BU datasets included the detection of 188 peptides containing sample-specific SNPs and 27 peptides crossing the junction of sample-specific novel splice junctions for each WHIM sample. In comparison, analysis of the TD datasets allowed the detection of 10 proteins containing WHIM-specific SNPs as shown in Table II. A single proteoform resulting from a WHIM-specific novel splice junction was also detected in both WHIM2 and WHIM16 samples, and its sequence coverage by TD appears in Supplemental Fig. S5. Since proteoforms containing sequences differing by one amino acid, for example, a reference protein sequence and a protein containing a cSNP, will likely coelute, TD was expected to be well suited for detecting allelic expression ratios. Indeed, as demonstrated in Fig. 2, gamma-synuclein (RefSeq:NP_003078, UniProt:O76070) and ribosomal protein L35 (RefSeq:NP_009140, UniProt:P42766) displayed that protein products from heterozygous alleles are being expressed at a roughly 1:1 ratio. In both cases, TD detected both protein forms and gave relative quantitative information about the abundance of the protein products resulting from the expression of the two different allelles. BU could provide that information only for gamma-synuclein, while in the case of ribosomal protein L35, peptides containing the site of the coding SNP were not detected.

**Table II:** Coding polymorphisms (cSNPs) detected and genotyped by TD proteomics

RefSeq	Uniprot accession	Protein description	cSNP	WHIM2	WHIM16
NP_000995	P05387	60S acidic RP P2	S64I	S64 and I64	S64
NP_001093162	Q6IS14	eIF-5A1-like	V137L	V137	V137 and L137
NP_001120865	P56378	6.8kDa mitochondrial proteolipid	I26V	I26 and V26	I26
NP_003078	O76070	γ-synuclein	E110V	E110	E110 and V110
NP_003854	O94777	DPM synthase subunit 2	T76S	N/D^a^	S76
NP_005013	P07737	Profilin-1	N10S	N10	N10 and S10
NP_006734	P98179	Putative RNA-binding protein 3	Y117D	Y117	Y117 and D117
NP_009140	P42766	RP L35	N101H	N101	N101 and H101
NP_037519	Q9UDW1	Cytochrome b-c1 complex subunit 9	I47V	I47 and V47	I47
NP_543011	Q96KR6	Protein FAM210B	P126S	P126 and S126	P126

^a^ not detected.

##### Label-Free Top-Down Quantitative Analysis

**Fig. 3.**
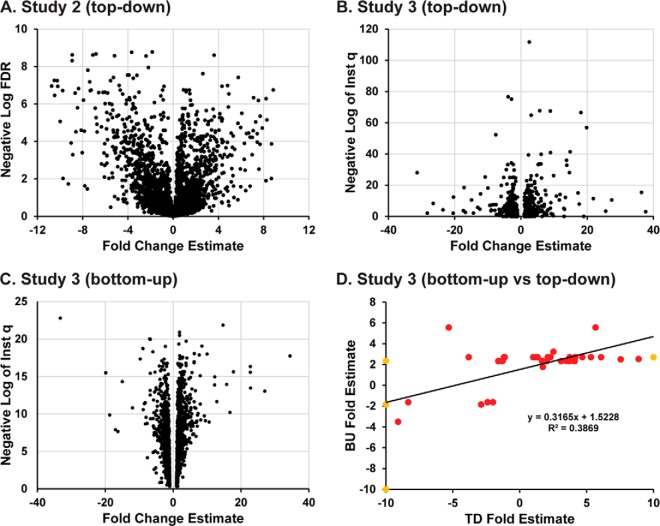
**Summary of quantitative results from Study 3.** (*A*) Volcano plot obtained using label-free TD quantitative analysis from comparison of 0–30kDa proteins in WHIM2 and WHIM16 (Study 2), (*B*) Volcano plot obtained using label-free TD quantitative analysis from comparison of 0–30kDa proteins in WHIM2 and WHIM16 (Study 3), (*C*) Volcano plot obtained using label-free BU quantitative analysis from comparison of 0–30kDa proteins in WHIM2 and WHIM16 (Study 3), (*D*) Correlation of BU and TD fold change estimates for significantly different entities.

Recently, a workflow for label-free top-down quantitation in discovery mode was described (11). Study 2 was designed to demonstrate the efficacy of this new method on the CompRef samples. In this scenario, a 2×3×6 study design (*i.e.* two states, three GELFrEE replicates, and six LC/MS injection technical replicates) was established for the comparative proteome analysis of WHIM2 and WHIM16. Proteins ranging from 0–30 kDa were isolated using GELFrEE followed by LC-MS/MS as described above. Next, a hierarchical linear model was applied to quantify intact proteoforms within the samples. A volcano plot (Fig. 3*A*) was generated, in which each proteoform was represented as a function of estimated effect size (in log_2_ fold-change) and the statistical confidence (FDR) that there was a difference in the normalized intensities between the two samples. Of the 5,975 quantitation mass targets detected in total, 1,031 of them were above the 5% FDR value comparing the WHIM2 and WHIM16 samples. Of all the quantitation mass targets, 538 were unambiguously identified using MS/MS information obtained during LC-MS. Among the differentially expressed proteoforms is the canonical isoform of gamma-synuclein (Fig. 2*A*), a protein known to be expressed in late-stage breast tumors (30). A list of all differentially expressed proteoforms from this study appears in Supplemental Table 3.

##### Comparison of TD and BU Label-Free Quantitation

In Study 3, the ability of TD and BU to quantify proteoform differential expression was evaluated. As described above, TD label-free quantitation is limited to the low MW proteome (<30 kDa). Briefly, GELFrEE separation of 0–30 kDa proteins into five discrete fractions was performed and fractions were run by both BU and TD methods. As such a direct comparison had not been achieved before, it is the trends and not the depth of proteome coverage that were of interest in the study, a design for which is shown in Supplemental Fig. S4. Volcano plots are shown (Figs. 3*B* and 3*C*) for the TD and BU results, respectively. Notice that the TD results have much greater spread in the fold change estimates but also that many proteoforms have much higher confidence in their differential expression, as represented by the correspondingly smaller instantaneous *q* values (the Y axis). This effect comes from proteoforms spanning multiple GELFrEE fractions and treating each fraction as a separate measure of the proteoforms' differential expression. For BU, 777,850 total spectra from 30 LC-MS/MS runs provided 49,185 uniquely identified peptides and a missing value percentage of 78.50%. For TD, a total of 4,950 quantitation mass targets were associated with proteoforms, with 67,434 MS1 observations used for quantification; 54.6% of the theoretically possible MS1 observations were missing. Since many proteoforms were found to elute across multiple GELFrEE fractions, only those mass groups that had been associated with proteoforms were quantified. The MS1 intensity values were standardized within fractions prior to the ANOVA.

When comparing the two proteomic techniques, it must be remembered that TD and BU proteomics measure different molecular entities. In comparing the differential results of the two techniques, there are a fixed number of distinct logical outcomes for a given protein. Both techniques can agree and show the identified protein to be either differentially expressed (DE) or not; the two techniques can disagree with one showing DE while the other does not, or one of the two techniques could have failed to observe the protein. All of these cases and the corresponding counts of proteins and proteoforms are shown in Table III. Notice first that there are 3,109 protein groups detected by BU that were not detected by TD, while only 64 proteins were uniquely detected by TD. This reflects the well-known advantage of BU in identifying large numbers of proteins present in a mixture. Nevertheless, TD provides a complementary look of the tumor proteome.

**Table III:** Overall quantitative results for Study 3 reveal a prevalence of concordant examples where proteoform-level changes differ substantially from that determined by BU

	Differentially expressed by TD	Not differentially expressed by TD	Not detected by TD
Differentially expressed by BU	12 proteins	14 proteins	314 proteins
	27 proteoforms	18 proteoforms	N/A
Not differentially expressed by BU	152 proteins	232 proteins	2,795 proteins
	233 proteoforms	584 proteoforms	N/A
Not detected by BU	0 proteins	64 proteins	
	0 proteoforms	99 proteoforms	

Top number are the RefSeq IDs detected in each cell, while the bottom number are the number of proteoforms detected.

TD often has more than one proteoform per RefSeq ID, and so the same ID may be in two or more boxes (as some proteoforms are differentially expressed, and others are not).

Of those proteins detected by both methods and mapping to the same RefSeq identifiers, the TD and BU quantitation agreed that there was differential expression at 60% of the time at the protein level. BU can only quantify what is happening on average to all proteoforms of a given protein due to prior proteolysis and not the individual proteoforms themselves. However, it is often the relative abundance of proteoforms harboring PTMs that changes and not the absolute abundance of the protein group. With regard to DE concordance, we found that many of the measurements agreed between the two methods. Disagreements in DE often arise from changes in PTM stoichiometry, creating dynamic behavior in TD proteomics, which is often obscured through peptide–protein inference in BU proteomics. Furthermore, the estimates of fold change between the two techniques agreed somewhat for those proteins and proteoforms where both methods agreed on differential expression (R-squared of 0.39, *r* = 0.62), as seen in Fig. 3*D*.

## DISCUSSION

Across all three studies comparing the WHIM samples, it is clear that BU proteomics is able to identify more proteins than TD. However, TD proteomics identifies and characterizes different entities than BU, namely intact proteoforms (*i.e.* the different molecular forms of a protein arising from a single gene). The number of proteoforms per RefSeq identification as discovered by TD varies significantly and the level of variation can be seen in Supplemental Fig. S6. Of note, only 21% of proteoforms are the sole representative of a RefSeq ID, and 52% of RefSeq IDs are seen by only one proteoform. The ability to detect and quantify proteoforms makes TD more sensitive at determining changes in PTMs and variant expression within complex samples that may be crucial in biological processes responsible for signal transduction.

**Fig. 4.**
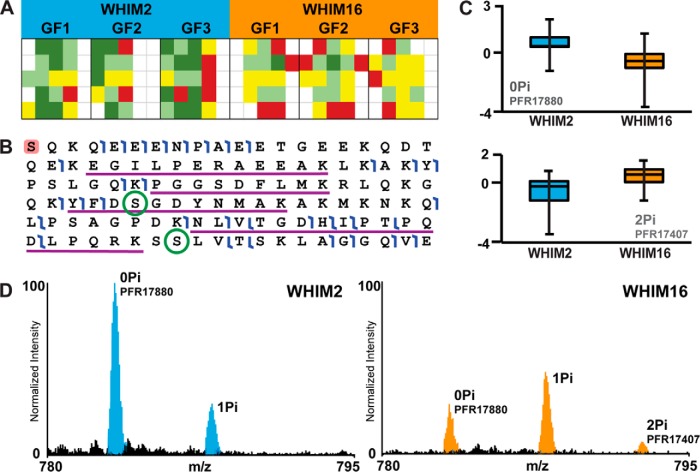
**Differential expression of alpha-endosulfine.** (*A*) Bottom-up heatmap illustrating number of alpha-endosulfine peptides identified in each replicate. Each row represents a separate peptide reporting uniquely on alpha-endosulfine, while columns in the map represent separate LC-MS/MS runs. Red represents one spectral count in the run, yellow two, light green three, and dark green four or more spectral counts. (*B*) Sequence of alpha-endosulfine including fragment ions (flags) detected by TD and peptide sequences (underlined) detected by BU. Two phosphorylation sites detected by TD are circled. The highlighted N-terminal amino acid indicates an N-terminal acetylation. (*C*) Boxplots illustrating abundance differences of alpha-endosulfine in WHIM2 (blue) and WHIM16 (orange) samples. The box in the boxplots show the median, first and third quartiles of all MS1 intensities detected for the protein. The bars show the range of the observed data. (*D*) Mass spectrum of alpha-endosulfine showing phosphorylation pattern changes of alpha-endosulfine in the two WHIM samples.

One clear example of these differences comes from alpha-endosulfine (RefSeq: NP_996929, UniProt:O43768) from Study 3, as shown in Fig. 4. It is known that phosphorylation of this protein affects its secondary structure and its corresponding protein–protein interactions (31). Both techniques found a greater abundance of the unmodified protein in WHIM2 (Figs. 4*A* and 4*C*). However, TD discovered a significant difference in phosphorylation stoichiometry that accompanied the abundance change and detected a diphosphorylated proteoform that was only present in WHIM16. Both methods had strong supporting evidence (Fig. 4*B*); TD confidently detected Isoform 1 of this protein with 121 observations of the unmodified form, and 48 observations on the diphosphorylated form, and BU detected the protein with five matched peptides covering 39.2% of the sequence and with 302 spectral counts. Although the BU analysis detected a peptide spanning one of the two phosphorylation sites, the analysis did not detect the phosphorylated peptide. As seen from Fig. 4*D*, the level of phosphorylation in WHIM16 is higher than in WHIM2, with both mono- and di- phosphorylated forms at higher relative abundance than in WHIM2, yet the unmodified form is higher in WHIM2. Despite the excellent sequence coverage by BU, it was not possible to capture this level of dynamism in PTM levels. Phosphopeptide enrichment prior to BU may have resulted in the detection of these phosphorylations, as shown in the analysis of similar xenografts (22). However, BU could not possibly report on the exact proteoform present, as co-occurrence information of PTMs is lost during proteolysis.

**Fig. 5.**
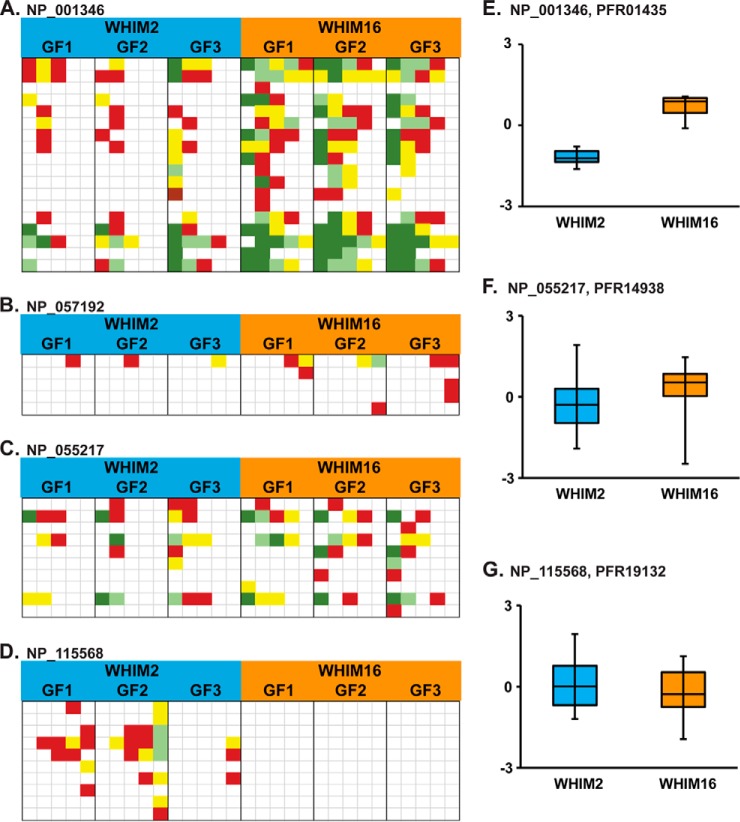
**Discordant examples of differential expression profiles as measured by BU and TD.** Panels *A–D* show heatmaps generated from BU spectral count data, while panels *E–G* contain corresponding boxplots from TD MS1 intensity data. Each row of the BU heatmaps represents a separate peptide reporting uniquely on the corresponding protein, while columns in the map represent separate LC-MS/MS runs. Red represents one spectral count in the run, yellow two, light green three, and dark green four or more spectral counts. The box in the boxplots show the median, first, and third quartiles of all MS1 intensities detected for the protein. The bars show the range of the observed data. Panels 5 *A* and *E* represent d-dopachrome decarboxylase (NP_001346) BU and TD, respectively; *C* and *F* represent cytochrome b-c1 complex subunit 8 (NP_055217), while *D* and *G* represent protein phosphatase 1 regulatory subunit 1B (NP_115568). Panel *B* shows BU data for androgen-induced gene 1 (NP_057192).

Now consider an example from those proteins that TD detected as differentially expressed but were not classified as such by the BU analysis. Figure 5*A* shows the results for d-dopachrome decarboxylase (RefSeq: NP_001346, UniProt: P30046), a protein in this class. Based on 24 MS1 observations and a strong characterization, TD data (Fig. 5*E*) showed this protein to be DE with an estimated fold change of over 36x more abundant in WHIM16 than WHIM2 (instantaneous *q* = 0.00001). The BU analysis had 91.5% sequence coverage from 18 different peptides and 786 spectral observations. The *t* test used to detect differential expression of the protein had a *p* value of .0086, but the critical value to maintain the 1% FDR for this test was .0019, thus, the protein was not considered DE by BU. Figure 5*B* shows the BU results for androgen-induced gene 1 (RefSeq: NP_057192, UniProt: Q9NVV5), a protein not detected by TD. The *t* test for this protein had a *p* score five ranks better than d-dopachrome decarboxylase, but it was identified by only five peptides, four of which were only seen once, and the other peptide had at most only three spectra in a single file. The effect of these marginal identifications is that they lengthen the list of identifications and force stronger identifications such as d-dopachrome decarboxylase to meet more stringent criteria in the DE analysis. This is an inherent tradeoff in quantitative omics studies. Less-stringent criteria can be used to accept more identifications, but in doing so, entities with less support are passed forward to the DE analysis. These less-well-supported entities, in this case BU protein IDs, increase the difference in treatment means needed for all entities detected to pass the multiple testing correction.

Decisions made in the data analysis pipeline can have a drastic effect on the sensitivity of either technique. Differences in the statistical criteria used in the DE analysis can cause a protein to be identified by BU as DE but not so by TD. An example is illustrated in the case of cytochrome b-c1 complex subunit 8 (RefSeq: NP_055217, UniProt: O14949) (Fig. 5*C*). The classification of DE hinges on the assumption of uniform variances in the log-transformed spectral counts. For the BU analysis, we assumed the log-transformed spectral counts between the WHIM2 and WHIM16 GELFrEE replicates had equal variance, and thus the corresponding increase in *p* value of the *t* test was sufficient to move this result onto the DE list for the BU study; if that assumption is relaxed, then the results are not sufficiently great for this protein to be considered DE, and the protein would be in agreement with the hierarchical linear model used for the TD analysis as not DE.

In some cases, the two methods can simply disagree as shown in the case of protein phosphatase 1 regulatory subunit 1B (RefSeq: NP_115568, UniProt: Q9UD71) (Fig. 5D). BU found 10 peptides each with one to three spectral counts in those LC runs that detected them in WHIM2, while no spectra were detected in WHIM16, leading to the conclusion that the protein is present in WHIM2 and absent in WHIM16. Meanwhile, TD had 33 observations, 12 from WHIM16 where they showed no difference in mean intensity (Fig. 5*G*). This case highlights the inferential problems that can arise from datasets containing many missing values. The BU dataset has 78.5% missing values. Therefore, when the WHIM2 data are near the detection threshold, *i.e.* no single file spotted any supporting peptide more than three times, the total absence of spectral counts from the WHIM16 LC-MS/MS runs is not necessarily compelling evidence of the protein not being expressed. In TD, it is easy to determine if the intensity of a proteoform is near the detection limit by looking at the signal-to-noise ratio of the intact measurement.

The difference in the ability of the two techniques to characterize major changes to proteins is highlighted by the response to cytoskeletal keratins. Keratin, in general, is an intermediate filament protein and one of the most common contaminants of proteomic studies. Like other intermediate filament proteins, keratins come in two complementary types, Type 1 and Type 2, and form polymeric complexes that shape both intracellular and extracellular structures (32). Type 2 intermediate filament proteins are known to have unique head and tail regions that differentiate their cellular function, while retaining the highly conserved central region responsible for forming the filamentous dimers (33). Forming the primary component of human hair and skin and containing a large and highly conserved middle rod section, keratins are frequently dismissed in MS-based proteomics as contaminants from sample preparation.

Type 2 cytoskeletal keratin 8 (K2C8, RefSeq: NP_002264, UniProt: P05787), however, is a cytoplasmic keratin used as a variable diagnostic tool in differentiating lobular and ductal breast cancers (34). Ductal carcinomas tend to stain diffusely positive for K2C8 markers (35), while lobular do not. In Study 3, the BU experiment found 98 peptides spanning nearly the entire length of K2C8 with 88.2% sequence coverage. The BU analysis found a strong increase of K2C8 in WHIM16 (1,727 *versus* 639 spectral counts; instantaneous *q* = 3.5 × 10^−5^; 2.7x fold increase in WHIM16). Unfortunately, because of the conserved nature of keratins, these results are easily dismissed as contamination. Panel *A* of Supplemental Fig. S7 shows that much of the BU data come from peptides unique to K2C8, and it is easy to see both its increase in WHIM 16 and a fairly uniform distribution of peptides across all three regions of the protein (head, rod, and tail).

By virtue of the “biomarker” search strategy (effectively a “no-enzyme” type search for TD data; see experimental section), the TD study found 17 proteoforms derived from K2C8, all of which were proteolytic fragments from the unique head or tail regions (see Supplemental Fig. S7). Eight out of 17 of the proteoforms were significantly increased in WHIM16 (ranging from 2–18-fold), the luminal-B cancer subtype. Two of the DE proteoforms from the N-terminal variable region contain phosphorylation, and one also contains a 3-hydroxy-l-proline. These proteoforms cannot be explained as hair or skin contamination as they represent unique sequences found only in the cytoplasmic keratin. Furthermore, all of the proteoforms from the head region end either one or two amino acids from S74, which is known to play an important role in keratin filament reorganization (35). These observations are consistent with increased proteolytic release of the head and tail domains of K2C8 in luminal *versus* basal PDX models. While cytokeratins and intermediate filaments have been identified as possible probes of breast cancer subtypes previously (33), these are the first such observations of head/tail proteolytic events only made possible via detection of intact proteoforms instead of tryptic peptides.

### 

#### 

##### Summary and Future Directions

While BU and TD generally display complementary sensitivities, the trends found within the data here provide a first tranche of specific observations. For example, BU identified 7.4 times as many proteins as TD, and 6.3 times as many proteins were found to be differentially expressed. TD proved sensitive for detecting proteoform-level differences below 30 kDa, such as the multiple phosphorylation forms of alpha-endosulfine, relative expression of heterozygous alleles like in gamma-synuclein or ribosomal protein L35, and domain-specific regions of keratin. BU discovered 10 times as many cancer–events but was not able to accurately predict which of these events were DE. While precise mapping of BU and TD data is complicated because they measure fundamentally different things, an early estimate of the proteoform-level dynamics not captured by BU can be made: For small, abundant proteins, changes in primary structure not captured by BU occur in about 40% of cases. Future quantitative TD studies will benefit from the analysis of larger proteins (30–60 kDa) as many proteins fall within that MW range. Given this study (and others), it is clear that there are significant benefits from the integration of BU and TD proteomics analyses, as a strong complementarity exists between peptide- and proteoform-level measurements.

## Supplementary Material

Supplemental Data

## Footnotes

^1^ The abbreviations used are:

BU: bottom up
CPTAC: Clinical Proteomic Tumor Analysis Consortium
DE: differential expression
GELFrEE: gel-eluted liquid fraction entrapment electrophoresis
K2C8: Type 2 cytoskeletal keratin 8
NGS: next-generation sequencing
PDX: patient-derived xenograft
PFR: proteoform record
PTM: posttranslational modification
SNP: single nucleotide polymorphism
TD: top down
WHIM: Washington University Human-in-Mouse
psi: pounds per square inch.
