# Supplementary material for: Integrated Bottom-Up and Top-Down Proteomics of Patient-Derived Breast Tumor Xenografts
Source: Mol Cell Proteomics. 2015 Oct 26;15(1):45–56. doi: 10.1074/mcp.M114.047480 (PMC4762530; doi:10.1074/mcp.M114.047480)
Supplement: Supplemental Data [file supp_15_1_45__index.html]

Integrated Bottom-up and Top-down Proteomics of Patient-derived Breast Tumor Xenografts — Integrated Bottom-Up and Top-Down Proteomics of Patient-Derived Breast Tumor Xenografts — Bottom-Up and Top-Down Proteomics of Breast Tumor Xenografts — Supplemental Data 

# Integrated Bottom-Up and Top-Down Proteomics of Patient-Derived Breast Tumor Xenografts

## Supplemental Data

- Supplemental Figures (.docx, 944 KB) - Supplemental Figures
- Supplemental Table 1 (.xlsx, 609 KB) - All Top Down Protein Identifications
- Supplemental Table 2 (.xlsx, 8.4 MB) - Bottom up protein identifications from Study 1
- Supplemental Table 3 (.xlsx, 322 KB) - Proteoform Quantitative data from TD runs on 8% GELFrEE fractions (Study 2)
- Supplemental Table 4 (.xlsx, 163 KB) - Proteoform Quantitative data from TD runs on 10% GELFrEE fractions (Study 3)
- Supplemental Table 5 (.xlsx, 3.5 MB) - Bottom up protein identifications and quantitative data from Study 3
