# Supplementary material for: Integrated Bottom-Up and Top-Down Proteomics of Patient-Derived Breast Tumor Xenografts
Source: Mol Cell Proteomics. 2015 Oct 26;15(1):45–56. doi: 10.1074/mcp.M114.047480 (PMC4762530; doi:10.1074/mcp.M114.047480)
Supplement: Supplemental Data [file 10.1074_M114.047480_mcp.M114.047480-1.docx]

**SUPPLEMENTAL DATA**

Supplemental Figure Legends

**Supplemental Fig. S1.** Silver stained gels for the visualization of representative GELFrEE separations for each of the studies described in Table 1. A) Study 1 used a 10% cartridge and 12 protein fractions were collected ranging in MW from 0 to 100 kDa, B) Study 2 used an 8% cartridge and proteins with MW<30kDa were collected in a single fraction, and C) Study 3 used a 10% cartridge and 5 protein fractions were collected ranging in MW from 0 to 30 kDa. **Supplemental Fig. S2.** Eight-step search tree used for the identification and characterization of proteoforms by TD proteomics.

**Supplemental Fig. S3.** Detailed schematic of how databases for searching TD data were constructed.

**Supplemental Fig. S4.** Detailed study designs for obtaining quantitative data for a) BU and b) TD used in Study 3.

**Supplemental Fig. S5.** Fragment map of proteoform containing a novel splice junction as identified by TD. Blue flags indicate the b- and y-ions detected.

**Supplemental Fig. S6.** Histogram showing the number of proteoforms per RefSeq ID as identified by TD proteomics.

**Supplemental Fig. S7.** K2C8 Keratin. Panel A: shows the spectral counts supporting each amino acid for WHIM16 (upper) and WHIM2 (lower). A) The central schematic (top) shows the extent of BU coverage on the Head, Rod, and Tail regions. Red regions denote amino acid sequences spanned by one or more non-proteotypic peptides, effectively regions shared with other Type II keratins. Panels B) and C) show the alignment of the proteoforms discovered by TD against the Head and Tail respectively. Green bars represent proteoforms that are statistically up in WHIM16. Pink dots show phosphorylation events seen on the proteoforms. The Purple circle in Panel B shows the location of the phosphorylation (S74) which has previously been shown to play an important role in keratin filament reorganization. Also note all proteoforms report N-terminal methionine cleavage for this protein, and proteoform 255608 is consistent with a previously unreported N-terminal acetylation (shown as a yellow circle). TD failed to identify any proteoforms from the central Rod region of the protein.

**Supplemental Fig. S1**

**
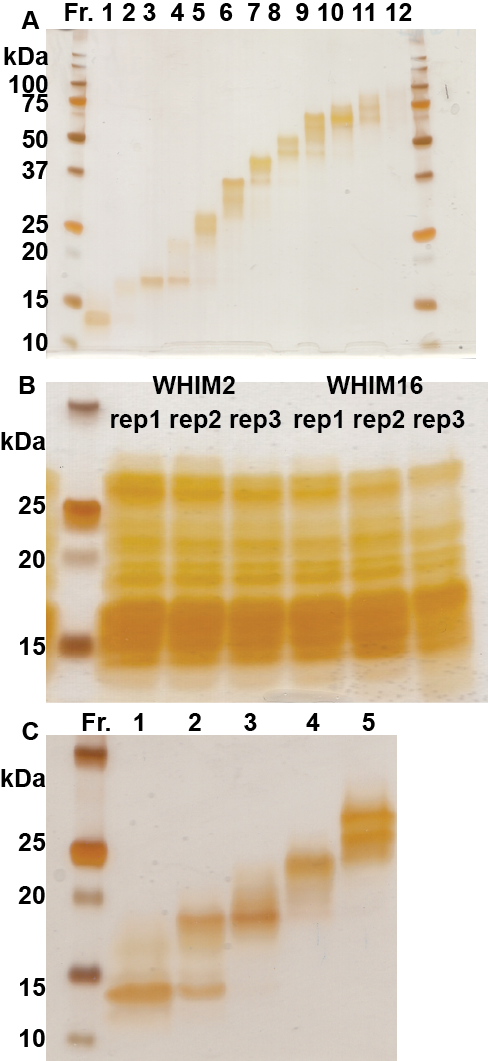
**

**Supplemental Fig. S2**


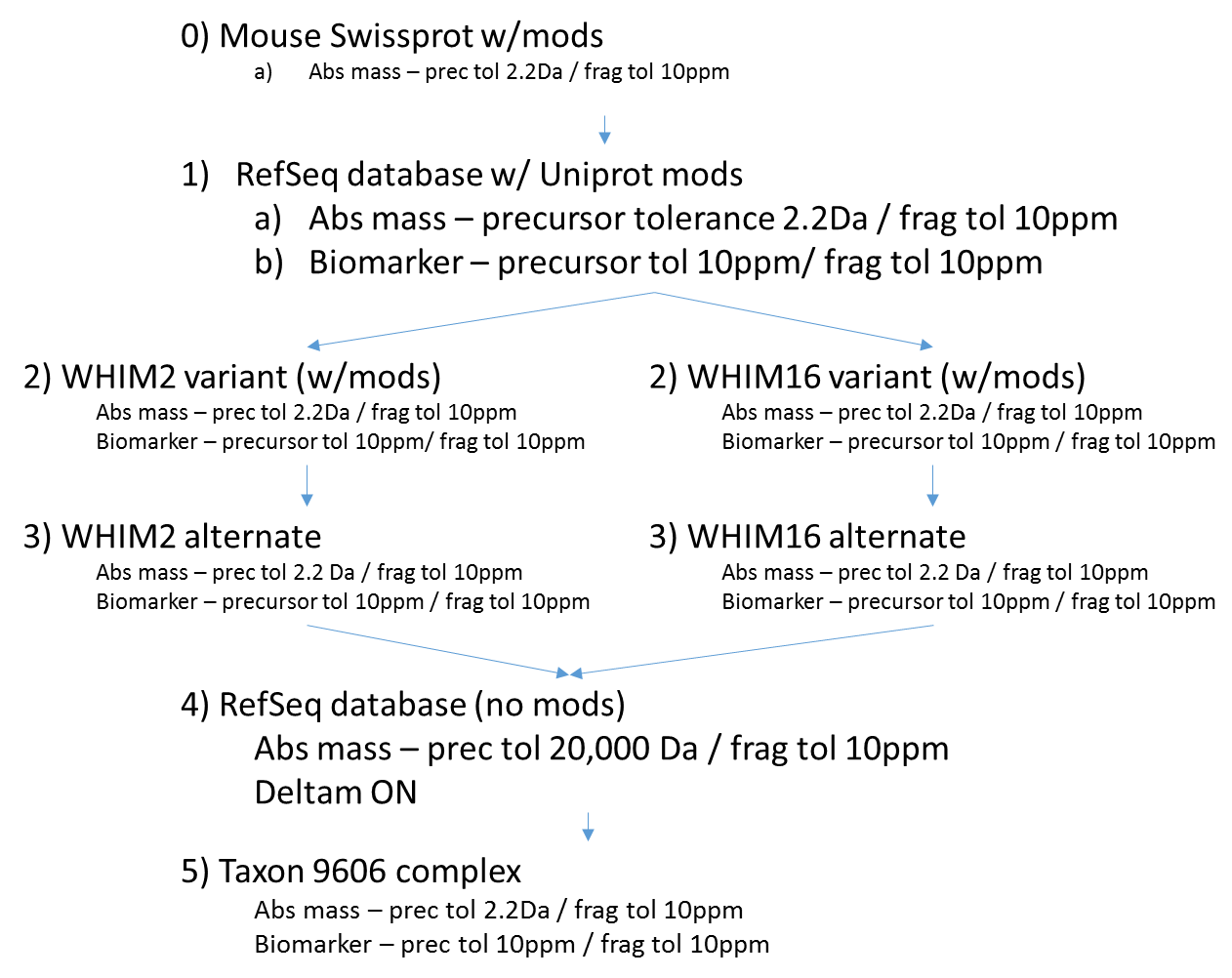


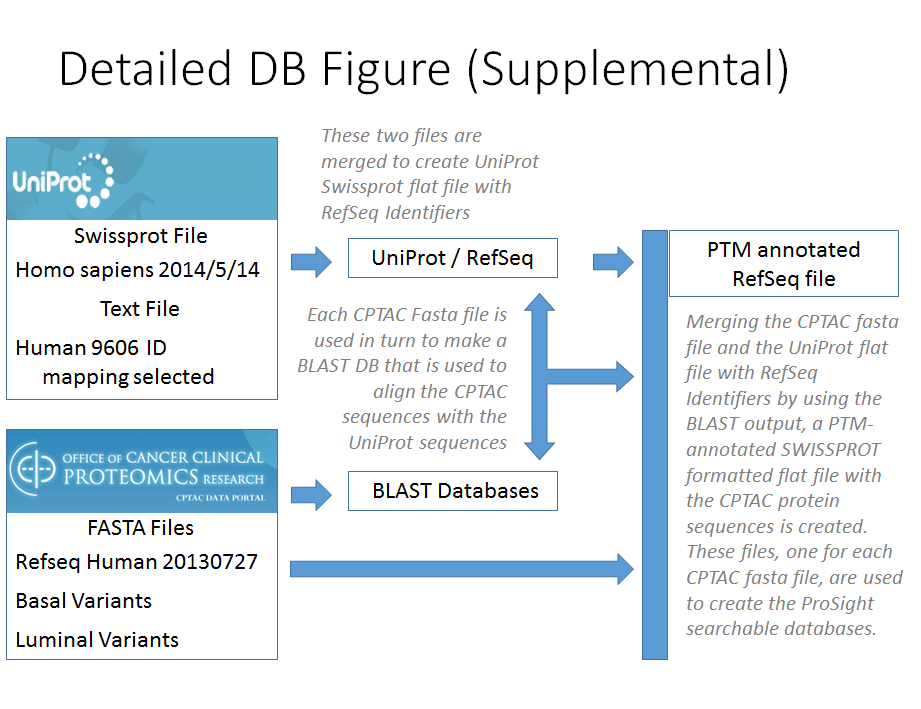
**Supplemental Fig. S3**

**Supplemental Fig. S4**


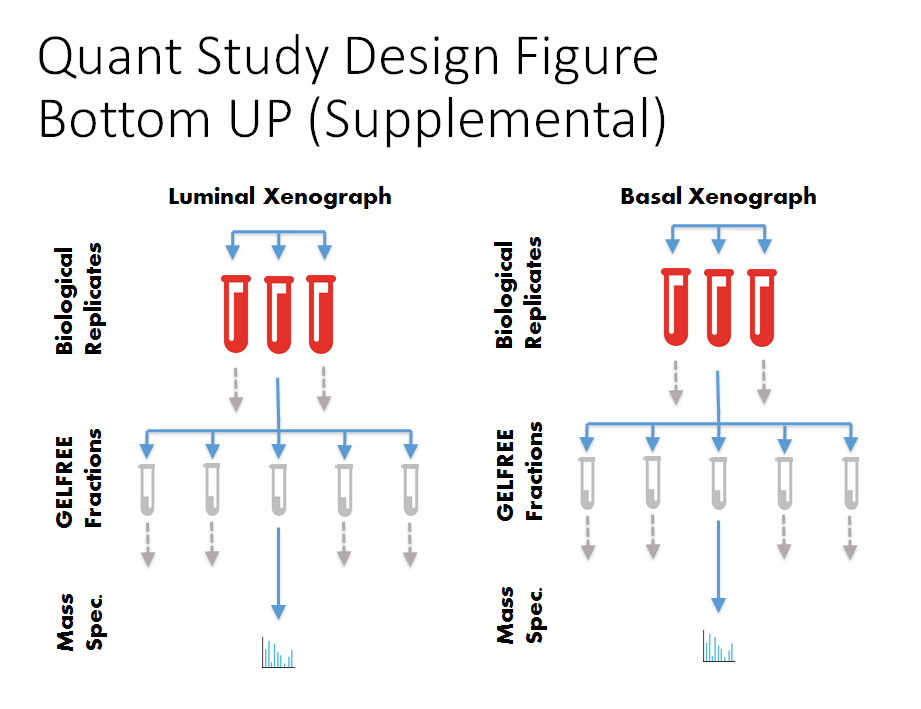


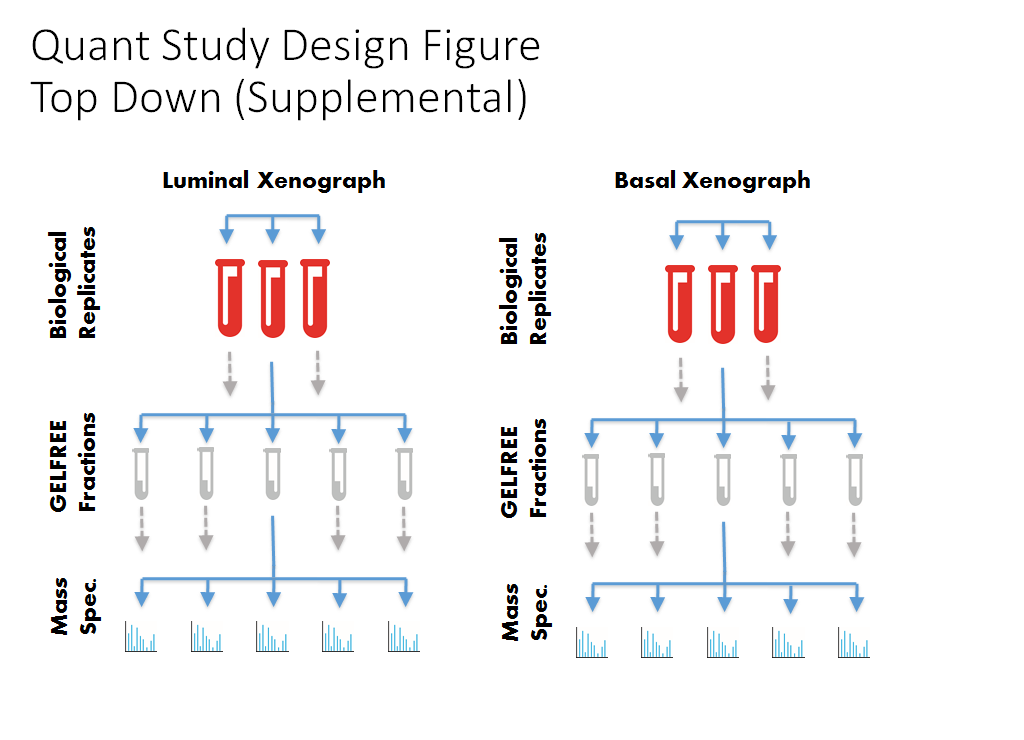


**Supplemental Fig. S5**

**
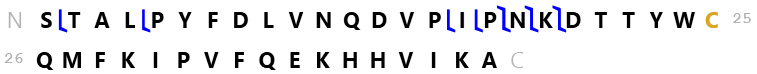
**

**Supplemental Fig. S6**

**Supplemental Fig. S7**

**
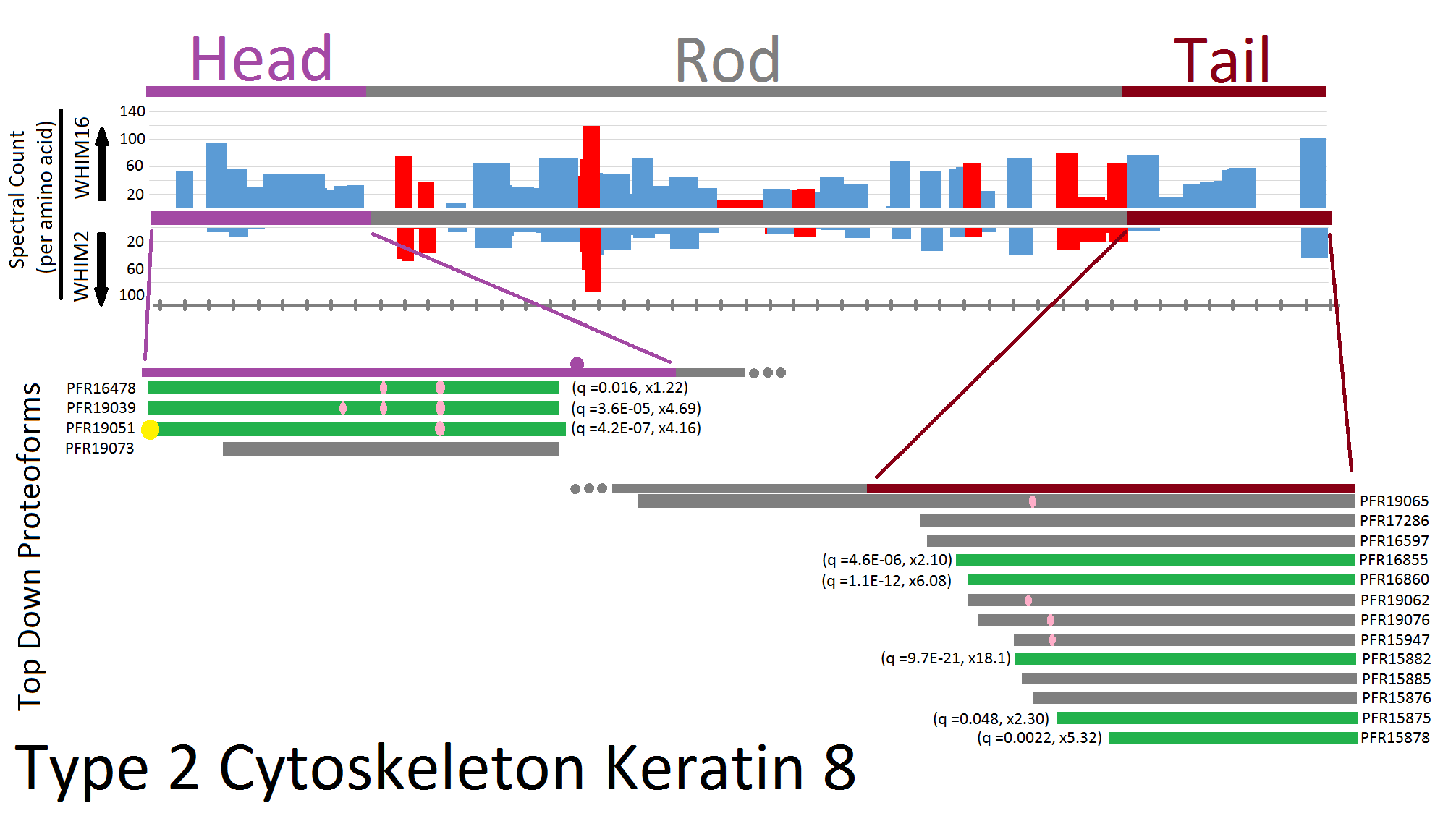
**

Supplemental Table 1. All Top Down Protein Identifications

Supplemental Table 2. Bottom up protein identifications from Study 1.

Sup.Table2.1.Peptides identified against RefSeq database – spectrum count

Sup.Table2.2.Proteins identified against RefSeq database

Sup.Table2.3. Identified peptides resulting from SNPs

Sup.Table2.4. Identified peptides resulting from NSJs

Supplemental Table 3. Proteoform Quantitative data from TD runs on 8% GELFrEE fractions (Study 2).

Supplemental Table 4. Proteoform Quantitative data from TD runs on 10% GELFrEE fractions (Study 3).

Supplemental Table 5. Bottom up protein identifications and quantitative data from Study 3.

Sup.Table5.1.Peptides identified against RefSeq database – spectrum count

Sup.Table5.2.Proteins identified against RefSeq database

Sup.Table5.3.Identified peptides resulting from SNPs

Sup.Table5.4.Identified peptides resulting from NSJs
